# Supplementary material for: Soft Selective Sweep on Chemosensory Genes Correlates with Ancestral Preference for Toxic Noni in a Specialist Drosophila Population
Source: Genes (Basel). 2020 Dec 29;12(1):32. doi: 10.3390/genes12010032 (PMC7824377; doi:10.3390/genes12010032)
Supplement: Supplementary file 1 [file genes-12-00032-s001.pdf]

```
#!/usr/bin/perl -w
#Pipeline of the population genomics analysis of chemosensory genes in Drosophila yakuba mayottensis. Each module (starting with ##) was run separately.
#use strict;
use List::Util qw(sum);

$cmd = "";
#Memory (in gigabytes) allocated to Picard
$mem = 8;

##Indexing the reference genome
#Path of reference genome fasta without .fasta
$reference = '';
#Path to minimap
$minimap = '';
#Path to samtools
$samtools = '';
#Path to all Picard java modules, which should all be in a single folder
$picard = '';

$cmd = $minimap."minimap2 -d " . $reference . ".mmi " . $reference . ".fasta";
system($cmd);
$cmd = "java -Xmx" . $mem . "g -jar " . $picard . " CreateSequenceDictionary REFERENCE=" . $reference . ".fasta    OUTPUT=" . $reference . ".dict";
system($cmd);
$cmd = "samtools faidx " . $reference . ".fasta";
system($cmd);

##Aligning Fastq reads to the reference genome
$i = 0;
#Path to the folder containing the *.fastq files
$genomes = '';
#List of the *.fastq files to be aligned. Remove the extension .fastq
@FastqFile = ();

for ($i = 0; $i < @FastqFile; $i++){
#Uncompress fastq files
   $cmd = 'gunzip ' . $genomes.$FastqFile[$i] . '_1.fastq';
   system($cmd);
   $cmd = 'gunzip ' . $genomes.$FastqFile[$i] . '_2.fastq';
   system($cmd);
#Create sam file
```

```
  $cmd = $minimap."minimap2 -ax sr -t 16 " . $reference . ".fasta "   . $genomes.$FastqFile[$i] . "_1.fastq "   . $genomes.$FastqFile[$i] . "_2.fastq -o " . $genomes.$FastqFile[$i] . ".sam";
  system($cmd);
#Create bam file
  $cmd = $samtools . "samtools view -bS " . $genomes.$FastqFile[$i] . ".sam > " . $genomes.$FastqFile[$i] . ".bam";
  system($cmd);
#Remove sam file
  $cmd = "rm " . $genomes.$FastqFile[$i] . ".sam";
  system($cmd);
#Compress fastq files
  $cmd = 'gzip ' . $genomes.$FastqFile[$i] . '_1.fastq';
  system($cmd);
  $cmd = 'gzip ' . $genomes.$FastqFile[$i] . '_2.fastq';
  system($cmd);
#Clean bam file, i.e. soft-clipping beyond-end-of-reference alignments and setting MAPQ to 0 for unmapped reads
  $cmd = "java -Xmx" . $mem . "g -jar " . $picard . "CleanSam INPUT=" . $genomes.$FastqFile[$i] . ".bam OUTPUT= " . $genomes.$FastqFile[$i] . "clean.bam";
  system($cmd);
#Remove bam file
  $cmd = "rm " . $genomes.$FastqFile[$i] . ".bam";
  system($cmd);
#Sort cleaned bam file by the reference sequence name (RNAME) field using the reference sequence dictionary (@SQ tag). Alignments within these subgroups are secondarily sorted using the left-most mapping position of the read (POS).
  $cmd = "java -Xmx" . $mem . "g -jar " . $picard . "SortSam SORT_ORDER=coordinate INPUT=" . $genomes.$FastqFile[$i] . "clean.bam OUTPUT=" . $genomes.$FastqFile[$i] . "sort.bam";
  system($cmd);
#Remove cleaned bam file
  $cmd = "rm " . $genomes.$FastqFile[$i] . "clean.bam";
  system($cmd);
  }

##Generating a synchronized file from sorted bam files
#use strict;

$i = 0;
#Abbreviation of the reference genome to be added at the beginning of the sync file name
$ref = "";
#Abbreviation of the population name if the multiple sorted bam files are from the same population
$pop= "";
#Path to PoPoolation2
```

```
$popoolation = '';
#Create the mpileup command
$cmd = "samtools mpileup -f " . $reference . ".fasta -B ";
push @cmd, $cmd;
for ($i = 0; $i < @FastqFile; $i++){
   push @cmd, $genomes.$FastqFile[$i] . " ";
   }
push @cmd, ">";
#Create the mpileup/sync output filename to be used in the mpileup and synchronizing commands
push @mp, $genomes;
push @mp, $ref."_";
push @mp, $pop.".mpileup";
$mp=join('',@mp);
push @cmd, $mp;
$cmd=join('',@cmd);
system($cmd);

#Create the synchronizing command
@cmd=();
@mp=();
$cmd = "java -ea -Xmx7g -jar " . $popoolation . "mpileup2sync.jar --input ";
push @cmd, $cmd;
push @cmd, $mp;
$cmd = " --output ";
push @cmd, $cmd;
@mp=split('\.',$mp);
push @cmd, $mp[0];
$cmd=".sync --fastq-type sanger --min-qual 20 --threads 8";
push @cmd,$cmd;
$cmd=join('',@cmd);
system($cmd);

##Convert read counts values in a sync file containing inbred lines into genotypes, for example 1145:1235:0:0:0:0 will become 1:1:0:0:0:0 for a heterozygous line and 21:0:0:0:0:0 will become 2:0:0:0:0:0 in a homozygous line for NY and CY (separately)
#Scaffolds to be retained, usually X, 2L, 2R, 3L and 3R
@Chr = ('X','2L','2R','3L','3R');#yakuba
#Scaffolds length according to the reference fasta or gff files (Note for simulans the length of the scaffolds in gff is +1 that of the reference fasta)
#@Chrsize=(21770863,22324452,21139217,24197627,28832112); #yakuba
#Cumulative value for each position according to the scaffolds order to be used in subsequent sort and join commands
@Chrcum=(0,21770863,44095315,65234532,89432159);#yakuba
```

```perl
#Define output sync file
my $outfile = ">".$genomes.$sync."_genotype.sync";
open(O,$outfile);
#Define output sync file for triallelic sites
my $outfile1 = ">".$genomes.$sync."_genotype_t.sync";
open(O1,$outfile1);
#Enter input sync file
my $File = $genomes.$sync.'.sync';
open(M, $File);
#Define the minimum depth at a site
$min=10;
#Define the minimum ratio of an allele
$mina=0.25;
#Parse the input file
while (<M>){
#Remove new line characters from the end of each line in the sync file
   chomp;
#Divide each line of the sync file into multiple elements (columns)
   @line=split('\t',$_);
   for($c=0;$c<@Chr;$c++){
#Retain the desired scaffolds defined in @Chr
       if($line[0] eq $Chr[$c]){
          @pos=();
#Push in the printable array (@pos) the cumulative value for the position
          $loc=$line[1]+$Chrcum[$c];
          push @pos, $loc;
#Then push the three first columns of the sync file, i.e. the scaffold, the position and the nucleotide at the reference genome
          for($l=0;$l<3;$l++){
             push @pos, $line[$l];
             }
#For each strain/population, note the first strain read counts is $line[3] and the last strain is $line[@line-1]
          for($p=3;$p<@line;$p++){
#Define the strain/population as an array @pop
             @pop=split(':',$line[$p]);
#Only retain the first four elements in @Nuc, i.e. A, T, C and G
             @strain=@pop[0..3];
#Do not genotype low-depth positions
             if(sum(@strain)<$min){
                push @pos, "0:0:0:0:$pop[4]:$pop[5]";
                }
#Genotype high-depth positions, alleles with read counts >= $mina
```

```
          else{
            $t=0;
            @genotype=();
            @homo=();
            $genotype='';
            for($b=0;$b<@strain;$b++){
              if(($strain[$b]/sum(@strain))>=$mina){
                push @genotype, 1;
                }
              else{
                push @genotype, 0;
                }
              }
#Homozygous sites
            if (sum(@genotype) == 1){
              @homo=(2*$genotype[0],2*$genotype[1],2*$genotype[2],2*$genotype[3]);
              push @homo, "$pop[4]:$pop[5]";
              $homo=join(':',@homo);
              push @pos, $homo;
               }
#Heterozygous sites
            if (sum(@genotype) == 2){
              push @genotype, "$pop[4]:$pop[5]";
              $genotype=join(':',@genotype);
              push @pos, $genotype;
               }
#Tri or quadri-allelic sites are omitted
            if (sum(@genotype) >2){
              $t++;
              push @pos, "0:0:0:0:$pop[4]:$pop[5]";
              }
            }
          }
        if($t > 0){
          print O1 "$_\n";
          }
        $pos=join("\t",@pos);
        print "$pos\n";
        print O "$pos\n";
        }
      }
    }
```

```
##For Mayotte flies sequences in two pools each with equal contributions from 11 different isofemale lines
#Name of the input sync file
$sync='';
#Define the size of the pools (i.e. chromosome numbers = 2N of pooled lines in diploids). In our case 22, since each pool consisted of females from 11 isofemale lines
@Pool=(22,22);
#Define output sync file
my $outfile = ">".$genomes.$sync."_genotype.sync";
open(O,$outfile);
#Define output sync file for triallelic sites
my $outfile1 = ">".$genomes.$sync."_genotype_t.sync";
open(O1,$outfile1);
#Enter input sync file
my $File = $genomes.$sync.'.sync';
open(M, $File);
#Define the minimum depth at a site
$min=10;
#Define the minimum ratio of an allele
$mina=0.25;
#Parse the input file
while (<M>){
#Remove new line characters from the end of each line in the sync file
   chomp;
#Divide each line of the sync file into multiple elements (columns)
#    @line=split("\t",$_);
   @line=split('\t',$_);
   for($c=0;$c<@Chr;$c++){
#Retain the desired scaffolds defined in @Chr
       if($line[0] eq $Chr[$c]){
          @pos=();
#Push in the printable array (@pos) the cumulative value for the position
          $loc=$line[1]+$Chrcum[$c];
          push @pos, $loc;
#Then push the three first columns of the sync file, i.e. the scaffold, the position and the nucleotide at the reference genome
          for($l=0;$l<3;$l++){
             push @pos, $line[$l];
             }
#For each strain/population, note the first strain read counts is $line[3] and the last strain is $line[@line-1]
          for($p=3;$p<@line;$p++){
#Define the strain/population as an array @pop
```

```
            @pop=split(':',$line[$p]);
#Only retain the first four elements in @Nuc, i.e. A, T, C and G
            @strain=@pop[0..3];
#Do not genotype low-depth positions
            if(sum(@strain)<$min){
               push @pos, "0:0:0:0:$pop[4]:$pop[5]";
               }
#Genotype high-depth positions, alleles with read counts >= 1/2N
            else{
               $t=0;
               @genotype=();
               $genotype='';
               for($b=0;$b<@strain;$b++){
                  if(($strain[$b]/sum(@strain))>=(1/$Pool[$p-3])){
                     if((($strain[$b]/sum(@strain))*($Pool[$p-3]))-0.5 >=
int(($strain[$b]/sum(@strain))*($Pool[$p-3]))){
                        push @genotype, int(($strain[$b]/sum(@strain))*($Pool[$p-3])) + 1;
                        }
                     else{
                        push @genotype, int(($strain[$b]/sum(@strain))*($Pool[$p-3]));
                        }
                     }
                  else{
                     push @genotype, 0;
                     }
                  }
               push @genotype, "$pop[4]:$pop[5]";
               $genotype=join(':',@genotype);
               push @pos, $genotype;
               }
            }
         $pos=join("\t",@pos);
         print "$pos\n";
         print O "$pos\n";
         }
      }
   }

##Merging multiple lines in a sync file into one
#Names of the input sync files
@sync=();
for($s=0;$s<@sync;$s++){
   my $outfile = ">".$genomes.$sync[$s]."_sum.sync";
```

```
    open(O,$outfile);
    my $File = $genomes[$s].$sync[$s].'_genotype.sync';
    open(M, $File);
    while (<M>){
        chomp;
        $A = 0;
        $T = 0;
        $C = 0;
        $G = 0;
        $N = 0;
        $gap = 0;
        @AoA=();
        @line=split("\t",$_);
        for($l=0;$l<4;$l++){
            print "$line[$l]\t";
            print O "$line[$l]\t";
            }
        $POP = @line - 4;
        for($p=4;$p<@line;$p++){
            @pop=split(":",$line[$p]);
            push @AoA, [@pop];
            }
        for($j=0;$j<$POP;$j++){
            $A = $AoA[$j][0]+$A;
            $T = $AoA[$j][1]+$T;
            $C = $AoA[$j][2]+$C;
            $G = $AoA[$j][3]+$G;
            $N = $AoA[$j][4]+$N;
            $gap = $AoA[$j][5]+$gap;
            }
        print "$A:$T:$C:$G:$N:$gap\n";
        print O "$A:$T:$C:$G:$N:$gap\n";
        }
    }

##Joining multiple *.sync files
#Paths to the folders containing the input *.sync files if all files are not in the same folder
#@genomes=();
#Names of the input sync files
@sync=();
#sort *.sync files according to the first column, i.e. the cumulative position value
for($s=0;$s<@sync;$s++){
    $cmd='sort -n '.$genomes.$sync[$s].'.sync >'.$genomes.$sync[$s].'_sort.sync';
```

```
    print "$cmd\n";
    system($cmd);
    }
```

#join sorted *.sync files according to the first column. Keep the first four columns from the first file and then add the fifth column progressively from each file

```
$File1=$sync[0].'_sort';
#$j=1;
for($s=1;$s<@sync;$s++){
    @cmd=();
    @out=();
    push @cmd, 'join -j 1 -o 1.1,1.2,1.3,1.4';
    for($i=5;$i<=4+$s;$i++){
        push @cmd, ',1.'.$i;
        }
    push @cmd, ',2.5 '.$genomes.$File1.'.sync '.$genomes.$sync[$s].'_sort.sync >'.$genomes;
    push @out, $ref;
    for($p=0;$p<=$s;$p++){
        @pop=split('_',$sync[$p]);
        push @out, '_'.$pop[1];
        }
    push @out, '_sort';
    $out=join('',@out);
    push @cmd, $out;
    push @cmd, '.sync';
    $cmd=join('',@cmd);
    print "$cmd\n";
    system($cmd);
    $File1=$out;
    }
```

##Estimating pi from sync files

```
#Name of the *_sort.sync file without the extension .sync
$sync='';
#Name of the output .pi file
my $outfile = ">".$genomes.$sync.".pi";
open(O,$outfile);
#Name of the input *_sort.sync file
my $infile = $genomes.$sync.".sync";
open(M, $infile);
#Parse the input file
while (<M>){
#Remove new line characters from the end of each line in the input file
```

```
   chomp;
#Divide each line of the sync file into multiple elements (columns) depending on space characters, e.g.,   , \t, \r, \n or \f. This is because the join command could substitute \r with
   @line=split('\s',$_);
#The sum of A, T, C, G alleles at a site for a population
   @size=();
#Sum of hereterozygote frequencies for all alleles at a site for each population
   @pi=();
#For each strain/population, note the first strain read counts is $line[4] and the last strain is $line[@line-1]
   for($p=4;$p<@line;$p++){
#Define the strain/population as an array @pop
       @pop=split(':',$line[$p]);
#Only retain the first four elements, i.e. A, T, C and G
       @strain=@pop[0..3];
#Estimate the population size, i.e. the sum of A, T, C and G
       $size=sum(@strain);
#Hereterozygote frequencies for all alleles at a site for a population
       @H=();
#Skip populations with coverage < 10 at a site
       unless($size < 10){
          for($n=0;$n<@strain;$n++){
#For each allele, estimate the frequency of drawing it (p) times the frequency of drawing an alternative allele (1 - p)
             $H=($strain[$n]/$size)*(1-($strain[$n]/$size));
             push @H, $H;
             }
#Heterozygosity frequency at a site for a population
          $pi=sum(@H);
          push @pi, $pi;
          }
       }
#Print the output
   if(scalar(@pi) == @line-4){
       print "$line[0]\t$line[1]\t$line[2]\t$line[3]";
       print O "$line[0]\t$line[1]\t$line[2]\t$line[3]";
       for($p=0;$p<@pi;$p++){
          print "\t$pi[$p]";
          print O "\t$pi[$p]";
          }
       print "\n";
       print O "\n";
       }
```

```
   }

##Estimate Hudson-Slatkin-Maddison (1992) Fst from sync file for population pairs
#Name of the *_sort.sync file without the extension .sync
$sync='';
#Name of the output .fst file
my $outfile = ">".$genomes.$sync.".fst";
open(O,$outfile);
#Name of the input *_sort.sync file
my $infile = $genomes.$sync.".sync";
open(M, $infile);
#Parse the input file
open(M, $infile);
while (<M>){
   chomp;
   @line=split('\s',$_);
   @size=();
   @A=();
   @T=();
   @C=();
   @G=();
   for($p=4;$p<@line;$p++){
      @pop=split(':',$line[$p]);
      @strain=@pop[0..3];
      $size=sum(@strain);
      push @size, $size;
#Create an array for population counts for each allele at a site
      unless($size < 10){
         push @A, $strain[0];
         push @T, $strain[1];
         push @C, $strain[2];
         push @G, $strain[3];
         }
      }
#Skip sites with any population with <10 reads
   if(scalar(@A) == @line-4){
#Estimate between-population heterozygosity, if all population are homozygous for the same allele,
$H = 0
      $H = 1 - ((sum(@A)/sum(@size))**2) - ((sum(@T)/sum(@size))**2) - ((sum(@C)/sum(@size))**2) -
((sum(@G)/sum(@size))**2);
#Retain only variable sites, i.e. $H>0
      if ($H > 0){
         print "$line[0]\t$line[1]\t$line[2]\t$line[3]";
```

```
        print O "$line[0]\t$line[1]\t$line[2]\t$line[3]";
#Compare pairs of populations
#Define allele frequencies in pop1
        for($i=0;$i<@line-4;$i++){
          @p1=($A[$i]/$size[$i],$T[$i]/$size[$i],$C[$i]/$size[$i],$G[$i]/$size[$i]);
          @q1=(1-($A[$i]/$size[$i]),1-($T[$i]/$size[$i]),1-($C[$i]/$size[$i]),1-($G[$i]/$size[$i]));
#Define allele frequencies in pop2
          for($j=$i+1; $j < @line-4; $j++){
            @Hw=();
            @Hb=();
            @p2=($A[$j]/$size[$j],$T[$j]/$size[$j],$C[$j]/$size[$j],$G[$j]/$size[$j]);
            @q2=(1-($A[$j]/$size[$j]),1-($T[$j]/$size[$j]),1-($C[$j]/$size[$j]),1-($G[$j]/$size[$j]));
            for($b=0;$b<@p1;$b++){
#For each allele, estimate average within-population heterozygosity and between-population heterozygosity in each pair of populations
              push @Hw, ($p1[$b] * $q1[$b]) + ($p2[$b] * $q2[$b]);
              push @Hb, ($p1[$b] * $q2[$b]) + ($p2[$b] * $q1[$b]);
              }
#Estimate average within-population heterozygosity for all alleles
            $Hw=sum(@Hw);
#Estimate between-population heterozygosity for all alleles
            $Hb=sum(@Hb);
#If the two populations are homozygous for the same allele (i.e. $Hb = 0), set $Fst = 0
            if($Hb==0){
              $Fst=0;
              }
#Estimate Fst
            else{
              $Fst=1-($Hw/$Hb);
              }
            print "\t$Fst";
            print O "\t$Fst";
            }
          }
          print "\n";
          print O "\n";
        }
      }
    }
exit;
```
